# Torque Teno Virus Viral Load as a Marker of Immune Function in Allogeneic Haematopoietic Stem Cell Transplantation Recipients

**DOI:** 10.3390/v12111292

**Published:** 2020-11-11

**Authors:** William Mouton, Anne Conrad, Antonin Bal, Mathilde Boccard, Christophe Malcus, Sophie Ducastelle-Lepretre, Marie Balsat, Fiorenza Barraco, Marie-Virginie Larcher, Gaëlle Fossard, Hélène Labussière-Wallet, Florence Ader, Karen Brengel-Pesce, Sophie Trouillet-Assant, Lyon HEMINF Study Group

**Affiliations:** 1 Joint Research Unit Hospices Civils de Lyon-bioMérieux, Civils Hospices of Lyon, Lyon Sud Hospital, 69310 Pierre-Bénite, France; antonin.bal@chu-lyon.fr (A.B.); mathilde.boccard@chu-lyon.fr (M.B.); karen.brengel-pesce@biomerieux.com (K.B.-P.); sophie.assant@chu-lyon.fr (S.T.-A.); 2 International Centre for Research in Infectiology (CIRI), INSERM U1111, CNRS UMR5308, ENS Lyon, Claude Bernard Lyon 1 University, 69364 Lyon, France; anne.conrad@chu-lyon.fr (A.C.); florence.ader@chu-lyon.fr (F.A.); 3 Infectious and Tropical Diseases Department, Civil Hospices of Lyon, Croix-Rousse Hospital, 69004 Lyon, France; 4 Virology and Human Pathology—Virpath Team, International Centre for Research in Infectiology (CIRI), INSERM U1111, CNRS UMR5308, ENS Lyon, Claude Bernard Lyon 1 University, 69008 Lyon, France; 5 Virology Laboratory, Institute of Infectious Agents, Civils Hospices of Lyon, Croix-Rousse Hospital, 69004 Lyon, France; 6 Immunology Laboratory, Civils Hospices of Lyon, Edouard Herriot Hospital, 69003 Lyon, France; christophe.malcus@chu-lyon.fr; 7 Clinical Haematology Department, Civils Hospices of Lyon, Lyon Sud Hospital, 69310 Pierre-Bénite, France; sophie.ducastelle-lepretre@chu-lyon.fr (S.D.-L.); marie.balsat@chu-lyon.fr (M.B.); fiorenza.barraco@chu-lyon.fr (F.B.); marie-virginie.larcher@chu-lyon.fr (M.-V.L.); gaelle.fossard@chu-lyon.fr (G.F.); helene.labussiere-wallet@chu-lyon.fr (H.L.-W.)

**Keywords:** allogeneic haematopoietic stem cell transplantation (allo-HSCT), torque teno virus (TTV), immune functional assay, immunocompetence, biomarker

## Abstract

Torque teno virus (TTV) has been proposed as a surrogate biomarker of T-cell function in allogeneic–haematopoietic–stem-cell transplantation (allo-HSCT). Conflicting data exists regarding the value of TTV to assess the degree of immunosuppression. The aim of the present study was to investigate the correlation between TTV viral load and immune function. Using samples from a prospective cohort composed of healthy-volunteers (HV) and allo-HSCT recipients at 6 months post-transplantation, we assessed the correlation between TTV viraemia and immune cell counts or T-cell proliferation capacity post-phytohaemagglutinin stimulation. TTV viraemia was detected in 68% of HV (*n* = 80) and 100% of allo-HSCT recipients (*n* = 41; *p* < 0.001); it was significantly higher in allo-HSCT recipients (3.9 vs. 2.1 Log copies/mL, *p* < 0.001). There was no correlation between T-cell function and CD3^+^T-cell count (rho: 0.002) suggesting that T-cell count can normalise without full functional recovery. Furthermore, no significant correlation was observed between TTV viraemia and absolute total/subset lymphocyte counts (rho: <0.13). The highest correlation was observed between TTV viral load and T-cell proliferation capacity (rho: −0.39). We therefore report an inverse correlation between T-cell function and TTV viraemia that is independent of T-cell count. Monitoring of TTV viraemia could be a fast suitable option to objectively assess the competence of immune function in at-risk populations.

## 1. Introduction

Allogeneic haematopoietic stem cell transplantation (allo-HSCT) is a cellular therapy used for the cure of malignant and non-malignant haematological disorders. From the immunological point of view, allo-HSCT recipients represent a highly specific population experiencing a phase of profound immunosuppression followed by gradual immune recovery [1,2,3]. Both the speed and quality of immune reconstitution are variable; these depend on haematological and transplant characteristics, as well as post-transplant complications such as graft-versus-host-disease (GvHD). This is also of importance as prolonged immunosuppression exposes allo-HSCT recipients to transplant rejection, relapse of the underlying disease and infectious complications [4].

During the immune reconstitution period, tools to effectively measure the individual degree of immunosuppression are needed. Absolute lymphocyte counts, such as counts of T-cell subtype populations, are currently used as biomarkers of immune reconstitution, although this is not informative of their function [5,6,7]. A simple blood biomarker mirroring T-cell function would be useful, and measuring the blood viral load of torque teno virus (TTV) may be of interest in this context. TTV is a ubiquitous, non-enveloped DNA virus from the *Anelloviridae* family with no known pathogenicity and constitutes the main component of the human blood virome [8]. Previous studies in the transplant setting have suggested that TTV viral load and/or the composition of the *Anelloviridae* virome could be surrogate markers of immune competence and could be useful to monitor patients’ immunity and to guide pharmacological interventions [9,10,11,12,13,14]. However, none of these analysed the correlation between TTV viral load and direct markers of immune function; only indirect markers of immunocompetence have been previously used, such as clinical adverse outcomes (type of disease, graft rejection, infections, or GvHD) or immune cell counts for which conflicting data exists, especially in the allo-HSCT context [9,15]. In addition to being based only on an indirect correlation, the link between TTV and immune function warrants further investigation. We therefore studied prospectively the correlation between TTV viraemia, immune cell counts and lymphocyte competency estimated by their proliferating capacity in allo-HSCT recipients at 6 months post-transplant.

## 2. Materials and Methods

### 2.1. Study Population

Adult (≥18 years) allo-HSCT recipients transplanted at the haematology department of the Lyon university hospital (France) and enrolled in the prospective, single-centre cohort study “VaccHemInf” between May 2018 and April 2020 were included. The VaccHemInf cohort study has been previously described and aims at studying vaccine immunogenicity and immune reconstitution after allo-HSCT [16]. The cohort has been approved by a regional review board (Comité de Protection des Personnes Sud-Est V, Grenoble, France; number 69HCL17_0769) and is registered in ClinicalTrial.gov (NCT03659773). At inclusion, demographics (age and sex), haematological and transplant-related (underlying haematological disease (based on 2016 revision of the World Health Organization classification of myeloid and lymphoid neoplasms), conditioning regimen, stem cell source, and donor type) as well as post-transplant characteristics (GvHD, immunomodulatory medication(s)) and immunosuppressive therapies including antithymocyte globulins, cyclosporine, tacrolimus, methotrexate, mycophenolate mofetil, cyclophosphamide, and corticosteroids ≥1 mg/kg >21 days) were retrieved from medical records through an electronic case report form (eCRF). The presence of viral opportunistic infection/reactivation (except TTV viral load) was also routinely monitored by quantitative Polymerase Chain Reaction (qPCR) in the routine virology laboratory and reported in the eCRF during the post-transplant period. Infections, infectious agent-related reactivations and diseases were reported using classification guidelines from European Group for Blood and Marrow Transplantation, as reported previously [16]. Concomitantly, healthy volunteers (HV) were recruited among donors to the Lyon blood transfusion centre (*Etablissement Français du Sang*, EFS) and were considered as a control group. According to the EFS procedures, informed consent was obtained from HV, and personal data were anonymous.

### 2.2. Blood Sampling

Before initiation of the recommended vaccination schedule after allo-HSCT [17,18], samples of heparinised whole blood and EDTA plasma were collected and processed for the measurement of quantitative and qualitative parameters of immune reconstitution.

### 2.3. TTV Viral Load Quantification

TTV viral DNA (elution volume 50 μL) was extracted from 200 μL of plasma sample using an easyMag extractor (bioMérieux, Marcy-l’Etoile, France) following the manufacturer’s instructions. The presence and viral load of TTV were then determined using the TTV R-GENE^®^ kit (available for research use only, not for diagnostic, Ref#69-030; bioMérieux, Marcy-l’Etoile, France) as previously described [19,20]. Log of TTV DNA copy number/mL (Log copies/mL) plasma are used to describe TTV viral load. The lowest viral load detected was 0.46 Log copies/mL.

### 2.4. T-Cell Proliferation Assay

Peripheral blood mononuclear cells (PBMCs) were isolated from fresh heparinised blood samples using a Ficoll density gradient centrifugation (U-04; Eurobio, Les Ulis, France). Then, 10^5^ cells/well were incubated for 24 h in supplemented culture medium (RPMI 1640; Eurobio) in a 96-well cell culture plate at 37 °C under 5% CO_2_. PBMCs were then stimulated in duplicate with phytohaemagglutinin (PHA) at 4 µg/mL (R30852801; Remel™, Dartford, Kent, UK) and incubated for 72  h. Then, cellular pellets were analysed for T-cell proliferation using the Click-It^®^ EdU AF488 flow kit (C10420; Life Technologies, Carlsbad, CA, USA) to measure incorporation of 5-ethynyl-2′-deoxyuridine (EdU) according to the previously published protocol [21]. The proportion (%) of EdU+ proliferating cells (among CD3^+^ T-cell) was obtained by flow cytometry analyses performed on a BD LSR Fortessa™ flow cytometer (BD Biosciences, San Jose, CA, USA). For each experiment, a minimum of 2.5 × 10^3^ CD3^+^ T-cells was recorded. Data were analysed using BD FACSDiva Software (version 8.0.3; BD Biosciences).

### 2.5. Post-Transplant T-Cell Immunophenotyping

Extensive T-cell immunophenotyping by flow cytometry on whole blood was performed at the immunology laboratory of the Lyon university hospital. Counts of naive CD4+ and CD8^+^ T-cell (CD45^+^CCR7^+^), central memory CD4^+^ and CD8^+^ T-cell (CD45RA^−^CCR7^+^), effector memory CD4^+^ and CD8^+^ T-cell (CD45RA^−^CCR7^+^), and differentiated memory CD4^+^ and CD8^+^ T-cell (CD45RA^+^CCR7^−^) were determined (cells/µL) as previously described [15]. The normal values for each type of cell have been provided by the immunology laboratory.

### 2.6. Statistical Analysis

The Shapiro–Wilk normality test was used to determine the distribution of data, and accordingly, quantitative data was expressed as mean (range) or median (interquartile range (IQR)). TTV viral load was log-transformed for analysis (Log copies/mL). For HV and allo-HSCT recipients’ datasets, analysis of variance was performed using *F*-test, and differences were calculated using a parametric unpaired t test with Welch’s correction. Correlations were assessed using a parametric Pearson rho correlation coefficient (rho (95% confidence interval, CI)). Regression analyses were conducted to evaluate the association between the dependent variable, TTV viral load, and independent variables (proportion of proliferating cells, absolute lymphocytes counts, and CD3^+^ T-cell counts). Differences in TTV plasma viral load according to several clinical characteristics were performed using the non-parametric Mann–Whitney test.

A *p*-value of < 0.05 was considered significant. Statistical analyses were conducted using GraphPad Prism^®^ software (version 5; GraphPad software, La Jolla, CA, USA) and R (version 3.5.1, R Core Team (2020). R: A language and environment for statistical computing. R Foundation for Statistical Computing, Vienna, Austria. (https://www.R-project.org/).

## 3. Results

### 3.1. Participant Characteristics

A total of 41 allo-HSCT recipients and 80 HV were included. Age (median (IQR): 46 (31–53) vs. 56 (40–64) years, *p* = 0.32) and sex ratio (1.6 vs. 1.4, *p* = 0.85) were similar among allo-HSCT recipients and HV. Allo-HSCT recipients were enrolled at a median (IQR) of 6 (5–8) months post-transplant. Patient characteristics are summarised below; of note, 17% (*n* = 7) had chronic GvHD and 78% (*n* = 32) of allo-HSCT recipients were still under immunosuppressive medication (Table 1).

### 3.2. Plasma TTV Viral Load in Allo-HSCT Recipients and HV

**Figure 1:**
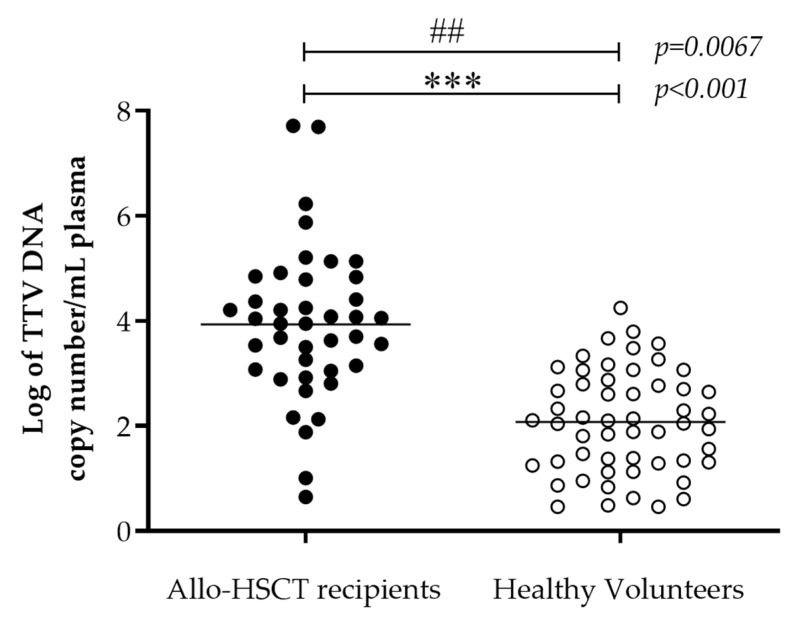
TTV viral load in healthy volunteers and allo-HSCT plasma samples. TTV viral load from 41 allo-HSCT recipients (black) and 54 healthy volunteers (white) plasma was quantified by real-time qPCR. Variance was compared using F-test (## *p* < 0.01). The mean TTV viral load (black line) was compared using unpaired t test with Welch’s correction (*** *p* < 0.001). Abbreviations: Allo. allogeneic; HSCT. haematopoietic stem cells transplantation; TTV. torque teno virus.

TTV was detected in 100% (41/41) of allo-HSCT recipients and 68% (54/80) of HV (*p* < 0.001). Mean (range) TTV viral load was significantly higher in allo-HSCT recipients compared to HV (3.9 (0.7–7.7) vs. 2.1 (0.5–4.3) Log copies/mL respectively, *p* < 0.001; Figure 1).

No correlation was found between the delay post-transplant (5–8 months) and plasma TTV viral load (Pearson’s rho ρ = 0.03, 95% CI (−0.2811 to 0.3338), *p* = 0.86; Figure S1).

### 3.3. Correlation between TTV Viral Load and T-Cell Counts or Proliferation Capacity

**Figure 2:**
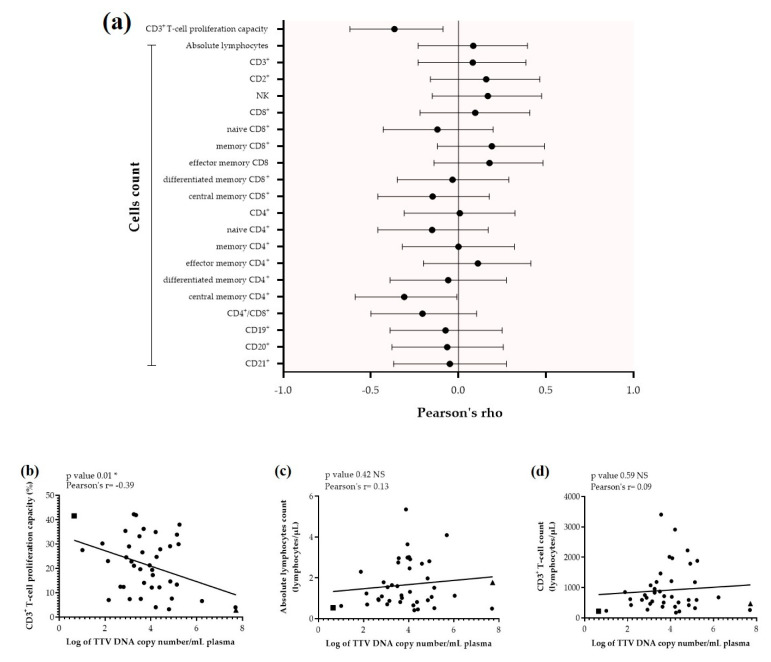
Overall correlation of TTV plasma viral load from 41 allo-HSCT recipients plasma versus T-cell subtype counts and CD3+ T-cell proliferation capacity (**a**). Pearson’s rho and 95% confidence interval (95% CI) for all parameters are represented by black dots and lines, respectively. Detailed correlation of TTV viral load from 41 allo-HSCT recipients’ plasma versus: (**b**) CD3+ T-cell proliferation capacity, (**c**) absolute lymphocyte count, and (**d**) CD3+ T-cell count. Extreme patients: “A” (square) and “B” (triangle), as well as the linear regression (black line) are presented. Correlation of TTV viral load (*x*-axis) and cell count or CD3+ T-cell proliferation capacity (*y*-axis) was determined using Pearson’s correlation coefficient. The asterisk (*) denotes statistical significance at p<0.05. Abbreviations: NK. Natural killer; PHA. Phytohemagglutinin; TTV. torque teno virus.

In allo-HSCT recipients, absolute lymphocytes and CD3^+^ T-cells counts were within normal value ranges. Five out of 10 lymphocyte subtype counts studied were lower than the normal value range, such as naïve and central memory CD3^+^CD4^+^ or CD3^+^CD8^+^ T-cells (individual values available in Table S1). A significant lower mean (range) proliferation capacity among CD3^+^ T-cells was observed in allo-HSCT recipients compared to HV (21.3% (2.9–42.3%) vs. 40.5% (29.7–55.3%), *p* < 0.001). In addition, a significantly wider range and a more heterogeneous distribution of proliferation capacity were observed among allo-HSCT recipients compared to HV (2.9–42.3% vs. 29.7–55.3%, respectively, F-test *p* = 0.004), indicating the inter-individual variability in the immune reconstitution after allo-HSCT (Figure S2). Correlations between TTV viral load and T-cell proliferation capacity or absolute cell counts were then analysed among allo-HSCT recipients (Figure 2A). The highest correlation was between TTV viral load and T-cell proliferation capacity (Pearson’s rho ρ = −0.39, (−0.62 to −0.09); Figure 2B); there was no significant correlation between TTV viral load and absolute lymphocytes (Figure 2C) or CD3^+^ T-cell counts (Figure 2D).

### 3.4. Correlation between (Post-)Transplant Characteristics and TTV Viral Load

**Figure 3:**
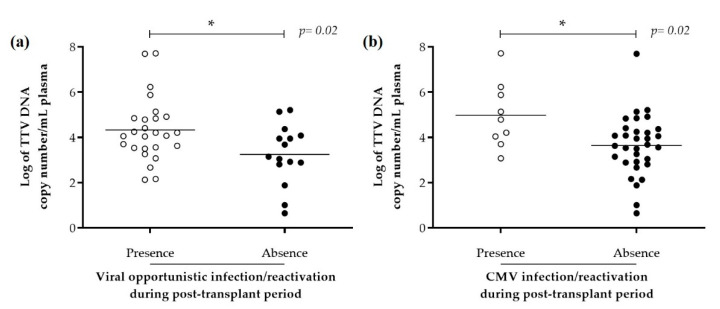
TTV viral load in allo-HSCT recipients according to post-transplant viral infection/reactivation. TTV viral load from 41 allo-HSCT with (white) or without (black) (**a**) viral opportunistic infection/reactivation and (**b**) CMV infection/reactivation during post-transplant period. Comparison between groups was performed using Mann–Whitney test, the asterisk (*) denotes statistical significance at *p* < 0.05. Abbreviations: Allo. allogeneic; CMV. Cytomegalovirus; HSCT. haematopoietic stem cells transplantation; TTV. torque teno virus.

There was a significantly higher median (IQR) plasma TTV viral load in allo-HSCT recipients with viral opportunistic infection/reactivation (4.1 Log copies/mL (3.5–4.9), *n* = 26, *p* = 0.02) compared to other allo-HSCT recipients (3.2 Log copies/mL (2.8–4.1), *n* = 15; Figure 3A). This was also the case for allo-HSCT recipients with CMV infection/reactivation (4.8 Log copies/mL (4.0–5.9), *n* = 9, *p* = 0.02) compared to other allo-HSCT recipients (3.7 Log copies/mL (2.9–4.3), *n* = 32; Figure 3B). Other characteristics did not significantly impact TTV viral loads in univariate analysis (Table S2).

### 3.5. Clinical Characteristics of Allo-HSCT Recipients with Extreme Values of TTV Viral Load

By analysing at an individual level the correlation between the TTV viral load and the T-cell proliferation capacity in response to PHA stimulation, we noticed that patient ‘A’ with the lowest viral load (0.65 Log copies/mL) had one of the highest T-cell proliferation capacities (41.6% EdU+ proliferating cells among CD3^+^ T-cell; square on Figure 2B). Conversely, patient ‘B’ with the highest viral load (7.72 Log copies/mL) had the lowest T-cell proliferation capacity (2.9% EdU+ proliferating cells among CD3^+^ T-cell; triangle on Figure 2B). Patient ‘A’ had an uncomplicated post-transplant course (Figure S3A); conversely, patient ‘B’ suffered from chronic GvHD (grade I requiring immunosuppressive therapy (topical corticosteroids), received monthly intravenous immunoglobulin infusions, and had presented multiple episodes of post-transplant infections (Figure S3B). Patient characteristics are summarised in Table 2.

## 4. Discussion

In the present study, we measured TTV viral load in allo-HSCT recipients at a median time of 6 months post-transplant and in HV. The frequency and level of TTV viral load in HV was coherent with the findings of a recent study reported by Focosi et al. (prevalence of 65% and 2.3 ± 0.7 Log copies/mL of TTV viral load) [22]. In allo-HSCT recipients, while absolute lymphocytes and CD3^+^ T-cells counts had normal values, TTV viral load was significantly higher than in HV, as previously reported [14,23]. Interestingly, despite heterogeneity in CD3^+^ T-cell proliferation among allo-HSCT recipients, the highest correlation was observed between TTV viral load and CD3^+^ T-cell proliferation in comparison with all T-cell subtype counts. Thus, as previously established, T-cell function and TTV viral load are inversely correlated [12,24]. In line with this observation, Focosi et al. suggested that TTV viral load is associated with the number of CD8^+^/CD57^+^ T-cells, a subtype of lymphocyte described as potential markers of immunosenescence and found in greater proportions in conditions such as acquired immunodeficiency states, transplants, or persistent viral infections [25].

In allo-HSCT recipients, the peak of TTV viral load occurs approximately at 3–6 months post-transplant before returning to the value observed prior to transplantation [9,26]. In the present study, we observed that at 6 months post-transplant during the immune reconstitution period, some patients are still unable to regulate the TTV replication, leading to high blood viral load, despite a sufficient number of T-cells. This observation has also been reported for other viruses such as cytomegalovirus (CMV) or Epstein–Barr virus (EBV) [9,27,28]

In addition, the lack of correlation between T-cell function, assessed by proliferative capacity in response to PHA stimulation, and the absolute number of T-cell subtypes (Pearson’s rho: 0.002, 95CI (−0.3257; 0.3223); data not shown) suggests that evaluation of lymphocyte counts at 6 months post-transplant cannot be used as a relevant marker of reconstitution of immune functions: T-cells can normalise without necessarily restoring immunity, and T-cells count does not allow to reveal the inter-individual variability of allo-HSCT recipients [7]. In addition, in the early post-transplant period, despite conflicting data [15], a few studies [13,29] report a weak correlation between T-cell count and TTV viral load. This correlation can be explained by the absolute lymphocyte count reconstitution post engraftment, which then serves as a TTV replication reservoir, thus avoiding extrapolation between TTV and T-cell function. Indeed, as suggested by Pradier et al., the correlation between T-cell subtype count and TTV viral load can vary according to the interval post-transplant [5]. This result highlights the need for new markers to evaluate lymphocyte function. The use of an immune functional assay (IFA) such as the assessment of post-stimulation T-cell proliferation capacity could fill this need but its implementation in clinical practice remains difficult. TTV could be interesting as a biomarker to estimate immune function and finally to predict infectious and/or clinical events related to the immune system [5,9,22,26,30].

The present study found a correlation between T-cell function, assessed by the proliferation capacity in response to PHA stimulation, and TTV viral load in a well-characterised cohort of allo-HSCT recipients. These data suggest an interplay between TTV replication and cellular immune reconstitution after transplantation, especially the reconstitution of T-cells, which are one of the main replicating sites of TTV [31].

The study does, however, have limitations, notably related to the small number of patients. For instance, because allo-HSCT recipients were referred for vaccination by their haematologists, we cannot exclude a selection bias, which could explain the absence of difference between clinical characteristics (e.g., underlying disease, conditioning, immunosuppressive therapy, or GvHD) and TTV viral load (Table S2). Moreover, it would be interesting to validate these results by assessing kinetics of TTV viral load and lymphocyte proliferative capacities at consecutive time points in this specific recipient population or in other immunocompromised populations. Furthermore, assessing the T-cell function through the measurement of their proliferating capacity might be a reductionist view of immune competency, and additional tests are needed to confirm the conclusions made.

## 5. Conclusions

In summary, we found an inverse correlation between the TTV viral load and T-cell function, independently of the count of T-cells. The evaluation of immune competency, for at-risk populations, using the monitoring of TTV viraemia, requires further assessment, as currently under evaluation in kidney transplant recipients [32]. Indeed, during the dynamic process of immune reconstitution post-transplant, a pivotal period for allo-HSCT recipients, a rapid and individual assessment of the immune system’s capacities could be of clinical interest to adapt anti-infectious prophylactic treatments, to guide the immunosuppressant treatment and in fine prevent graft rejection GvHD, infectious events and relapsing disease, which are the main causes of morbimortality in this population.

## Figures and Tables

**Table 1:** Clinical characteristics of allogeneic haematopoietic stem cell transplant recipients.

	Patients *n* = 41
Demographics	56 (40–64)
Age, median (IQR]	24 (59)
Time from transplantation in months, median (IQR)	6 (5–8)
Haematological and transplant-related characteristics. n (%)	
Underlying haematological diseases	
Myeloid neoplasm and acute leukaemia	37 (90)
Mature lymphoid. histiocytic. and dendritic neoplasms	4 (10)
CR before the engraftment. n (%)	39 (95)
Donor types	
Matched related	23 (56)
Unrelated	18 (44)
Fully matched	15 (37)
HLA mismatched	3 (7)
Stem cell source	
Peripheral blood cells	28 (68)
Bone marrow	13 (32)
Conditioning regimen	
MAC	17 (41)
RIC	24 (59)
TBI	11 (27)
Post-transplant complications. n (%)	
Acute GvHD	30 (73)
Grade I/II	21/9
Chronic GvHD	7 (17)
Limited/Extensive	5/2
Immunophenotyping. mean (range)	
Absolute lymphocytes (NV. 1000–2800/µL)	1659 (410–5350)
CD3^+^ T-cells (NV. 521–1772/µL)	915 (175–3406)
CD3^+^ CD4^+^ T-cells (NV. 336–1126/µL)	273 (38–876)
Naïve CD4^+^ (CD45^+^CCR7^+^) (NV. 121–456/µL)	45 (0–445)
Central Memory CD4^+^ (CD45RA^−^CCR7^+^) (NV. 92–341/µL)	60 (1–168)
Effector Memory CD4^+^ (CD45RA^−^CCR7^−^) (NV. 59–321/µL)	163 (4–522)
Differentiated Memory CD4^+^ (CD45RA^+^CCR7^−^) (NV. 11–102/µL)	19 (0–147)
CD3^+^ CD8^+^ T-cells (NV. 125–780/µL)	602 (60–2779)
Naïve CD8^+^ (CD45^+^CCR7^+^) (NV. 86–257µL)	40 (0–241)
Central Memory CD8^+^ (CD45RA^−^CCR7^+^) (NV. 19–93/µL)	17 (0–127)
Effector Memory CD8^+^ (CD45RA^−^CCR7^−^) (NV. 15–162/µL)	286 (0–1517)
Differentiated Memory CD8^+^ (CD45RA^+^CCR7^−^) (NV. 39–212/µL)	257 (0–1474)
CD4^+^/CD8^+^ ratio (NV. 0.9–6)	0.84 (0.13–8.88)
Post-transplant immunomodulatory therapy at inclusion, n (%)	
IS Therapy	32 (78)
Corticosteroids	5 (12)
IVIG infusion	23 (56)
Time since last IVIG infusion, month (median (IQR))	4 (2–5)
DLI	7 (17)
ECP	2 (5)

Abbreviations: Allo. allogeneic; CR. complete remission; DLI. donor lymphocyte infusion; ECP. extracorporeal photochemotherapy; GvHD. graft-versus-host disease; HLA. human leukocyte antigen; HSCT. haematopoietic stem cells transplantation; IQR. interquartile range; IS. immunosuppressive; MAC. myeloablative conditioning; NK. Natural Killer; NV. normal values; RIC. reduced intensity conditioning; TBI. total body irradiation.

**Table 2:** Clinical characteristics of patients with extreme values of TTV viral load.

	Patient A	Patient B
Demographics		
Age	52	57
Sex	Male	Female
Haematological and transplant-related characteristics		
Underlying haematological diseases	Mature B cells neoplasm	Acute myeloid leukaemia
CR before the engraftment	Yes	Yes
Donor types	Geno-identical	Pheno-identical (9/10)
Stem cell sources	Bone marrow	Peripheral blood cells
Conditioning regimen	MAC	RIC
Total body irradiation	Yes	No
Post-transplant complications		
Acute GvHD	No	Yes. grade I
Chronic GvHD	No	No
Post-transplant immunomodulatory therapy		
IS therapy	No	Yes
IVIG infusion	No	Yes
Number of infections after transplantation	0	5
TTV viral load in Log copies/mL	0.65	7.72
CD3^+^ T-cell proliferation capacity	41.6	2.9

Abbreviations: GvHD. graft versus host disease; IS. immunosuppressive therapy; IVIG; intravenous immunoglobulin; MAC. myeloablative conditioning; RIC. reduced intensity conditioning; TTV. torque teno virus.

## Supplementary Materials

The following are available online at https://www.mdpi.com/1999-4915/12/11/1292/s1, Figure S1: Correlation between TTV viral load and delays from HSCT; Figure S2: Proliferation capacity of CD3^+^ T-cell of allo-HSCT recipients compared to healthy volunteers; Figure S3: Chronological descriptive follow-up of patients with extreme values of TTV viral load; Table S1: T-cell immunophenotyping of allogeneic haematopoietic stem cell transplant recipients and Table S2: Comparison of TTV plasma viral load with clinical characteristics of 41 allogeneic haematopoietic stem cell transplant recipients.
