# Supplementary material for: Torque Teno Virus Viral Load as a Marker of Immune Function in Allogeneic Haematopoietic Stem Cell Transplantation Recipients
Source: Viruses. 2020 Nov 11;12(11):1292. doi: 10.3390/v12111292 (PMC7696489; doi:10.3390/v12111292)
Supplement: Supplementary file 1 [file viruses-12-01292-s001.zip › viruses-980283-supplementary/Supplementary Figures.pdf]

viruses

MDPI

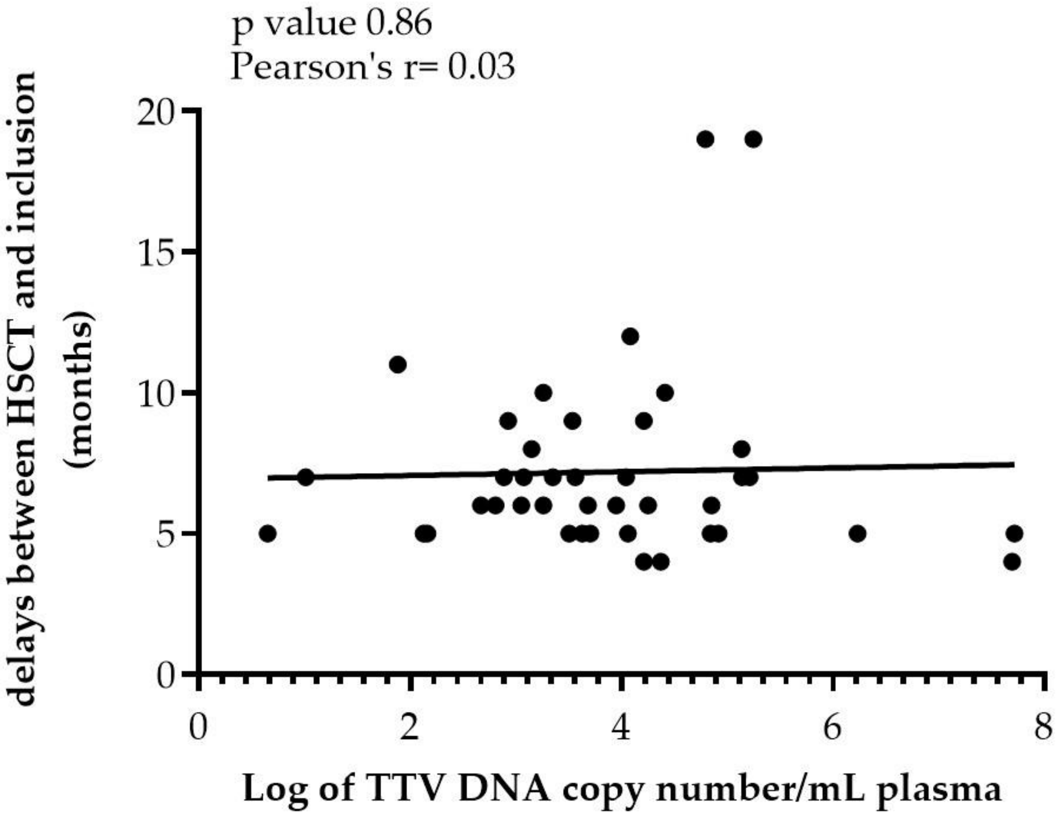

Figure S1. Correlation between TTV viral load and delays from HSCT. Detailed correlation of TTV plasma viral load expressed in Log of TTV DNA copy number /mL plasma from 41 allo-HSCT recipients plasma versus delays in month between HSCT and inclusion. Correlation of TTV viral load (x-axis) and delays (y-axis) was determined using Pearson's correlation coefficient. Linear regression is represented by a black line. Abbreviations: HSCT. haematopoietic stem cells transplantation; TTV. torque teno virus.

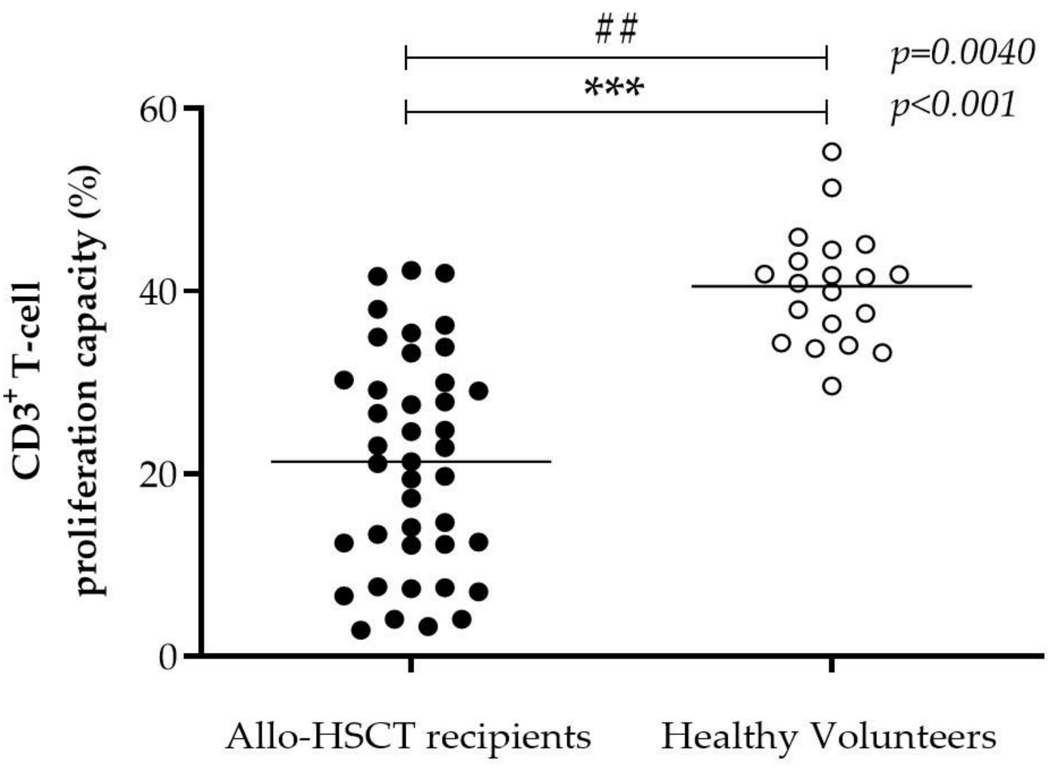

Figure S2. Proliferation capacity of CD3+ T-cell of allo-HSCT recipients compared to healthy volunteers.CD3+ T-cell proliferation capacity of 41 allo-HSCT recipients (black) and 20 HV (white) was quantified after a 3 days stimulation with a mitogen (PHA) and measured by flow cytometry using Click-It® EdU AF488 flow kit.Variance of both population was compared using F-test (##. p<0.01). Comparison mean (black line) was performed using unpaired t test with Welch's correction (***. p< 0.001). Abbreviations: Allo. allogeneic; HSCT. haematopoietic stem cells transplantation; HV. healthy volunteers.

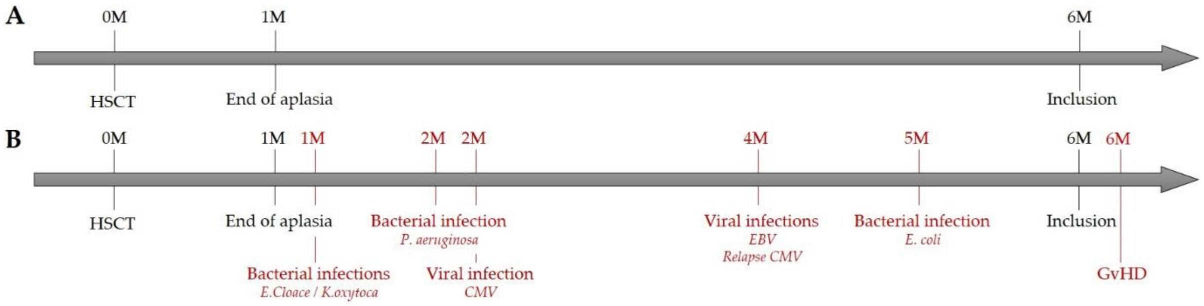

Figure S3. Chronological descriptive follow-up of patients with extreme values of TTV viral load. Timeline description of the main clinical steps (in black) and infectious episodes (in red) between transplantation and inclusion for patient "A" and patient "B". The patient "A" had the lowest TTV viral load and conversely the patient "B" the highest TTV viral load. Abbreviations: CMV. Cytomegalovirus; EBV. Epstein-Barr virus; GvHD. graft versus host disease; HSCT. Haematopoietic stem cell transplantation; M. Month..
