# Supplementary material for: Torque Teno Virus Viral Load as a Marker of Immune Function in Allogeneic Haematopoietic Stem Cell Transplantation Recipients
Source: Viruses. 2020 Nov 11;12(11):1292. doi: 10.3390/v12111292 (PMC7696489; doi:10.3390/v12111292)
Supplement: Supplementary file 1 [file viruses-12-01292-s001.zip › viruses-980283-supplementary/Table S1- R1.pdf]

Table S1. T-cell immunophenotyping of allogeneic haematopoietic stem cell transplant recipients. Abbreviations: NV. normal values.

Patients	Absolute lymphocytes NV. 1000-2800/μL	$CD3^+$ T-cells NV. 521-1772/μL	$CD3^+$ $CD4^+$ T-cells NV. 336-1126/μL	Naïve $CD4^+$ ($CD45^+CCR7^+$) NV. 121-456/μL	Central Memory $CD4^+$ ($CD45RA^-CCR7^+$) NV. 92-341/μL	Effector Memory $CD4^+$ ($CD45RA^-CCR7^-$) NV. 59-321/μL	Differentiated Memory $CD4^+$ ($CD45RA^+CCR7^-$) NV. 11-102/μL	$CD3^+$ $CD8^+$ T-cells NV. 125-780/μL	Naïve $CD8^+$ ($CD45^+CCR7^+$) NV. 86-257μL	Central Memory $CD8^+$ ($CD45RA^-CCR7^+$) NV. 19-93/μL	Effector Memory $CD8^+$ ($CD45RA^-CCR7^-$) NV. 15-162/μL	Differentiated Memory $CD8^+$ ($CD45RA^+CCR7^-$) NV. 39-212/μL	$CD4^+/CD8^+$ ratio NV. 0.9-6
AL001	950,00	273,00	122,00	13,00	27,00	82,00	4,00	134,00	15,00	5,00	38,00	75,00	0,91
AL002	2690,00	2224,00	391,00	14,00	46,00	334,00	0,00	1817,00	18,00	45,00	1259,00	497,00	0,22
AL003	4090,00	1880,00	876,00	445,00	74,00	360,00	52,00	979,00	241,00	0,00	288,00	447,00	0,89
AL004	5350,00	1969,00	819,00	223,00	74,00	522,00	147,00	1182,00	51,00	0,00	384,00	739,00	0,69
AL005	1060,00	592,00	533,00	85,00	80,00	368,00	73,00	60,00	9,00	4,00	31,00	16,00	8,88
AL006	1120,00	679,00	115,00	4,00	29,00	82,00	0,00	562,00	8,00	0,00	247,00	304,00	0,20
AL007	3010,00	2910,00	258,00	74,00	23,00	162,00	66,00	1994,00	46,00	0,00	467,00	1474,00	0,13
AL008	2750,00	1465,00	315,00	43,00	86,00	186,00	14,00	1140,00	208,00	127,00	505,00	301,00	0,28
AL009	1590,00	872,00	261,00	19,00	66,00	176,00	0,00	456,00	67,00	7,00	259,00	122,00	0,57
AL010	810,00	369,00	206,00	33,00	75,00	98,00	0,00	193,00	49,00	27,00	100,00	16,00	1,07
AL011	1640,00	832,00	293,00	51,00	85,00	157,00	17,00	516,00	38,00	17,00	347,00	114,00	0,57
AL012	1770,00	467,00	126,00	0,00	11,00	115,00	0,00	326,00	0,00	7,00	299,00	19,00	0,39
AL013	1780,00	666,00	270,00	33,00	109,00	129,00	4,00	364,00	26,00	9,00	242,00	87,00	0,74
AL014	650,00	368,00	119,00	1,00	21,00	97,00	1,00	230,00	29,00	10,00	82,00	110,00	0,52
AL015	2460,00	1212,00	314,00	59,00	91,00	165,00	29,00	909,00	77,00	81,00	180,00	572,00	0,35
AL016	1230,00	618,00	173,00	6,00	37,00	130,00	2,00	427,00	10,00	7,00	140,00	270,00	0,40
AL017	530,00	222,00	151,00	10,00	136,00	4,00	0,00	73,00	22,00	49,00	1,00	0,00	2,09
AL018	3640,00	2011,00	287,00	44,00	76,00	167,00	6,00	1586,00	49,00	0,00	771,00	761,00	0,18
AL019	2910,00	959,00	356,00	35,00	141,00	180,00	0,00	585,00	92,00	31,00	373,00	89,00	0,61
AL020	1540,00	1075,00	259,00	39,00	57,00	164,00	15,00	790,00	15,00	0,00	437,00	332,00	0,33
AL021	1090,00	752,00	207,00	62,00	29,00	115,00	44,00	573,00	12,00	0,00	197,00	362,00	0,36
AL022	1970,00	591,00	334,00	45,00	81,00	209,00	0,00	225,00	44,00	17,00	117,00	48,00	1,49
AL023	1140,00	507,00	321,00	53,00	129,00	141,00	9,00	149,00	14,00	4,00	57,00	74,00	2,16
AL024	1010,00	717,00	317,00	17,00	67,00	232,00	13,00	389,00	64,00	15,00	129,00	181,00	0,82
AL025	490,00	262,00	38,00	0,00	16,00	22,00	0,00	218,00	4,00	12,00	200,00	0,00	0,17
AL026	1510,00	1178,00	218,00	0,00	23,00	194,00	0,00	955,00	0,00	0,00	895,00	49,00	0,23
AL027	2810,00	1789,00	559,00	106,00	50,00	403,00	95,00	1252,00	18,00	18,00	662,00	554,00	0,45
AL028	810,00	513,00	183,00	14,00	40,00	129,00	4,00	337,00	10,00	10,00	218,00	99,00	0,54
AL029	510,00	321,00	85,00	71,00	1,00	13,00	67,00	227,00	0,00	0,00	0,00	225,00	0,37
AL030	2300,00	853,00	432,00	38,00	168,00	226,00		364,00	76,00	51,00	165,00	72,00	1,19
AL031	450,00	216,00	75,00	5,00	36,00	35,00		129,00	58,00	7,00	40,00	25,00	0,58
AL032	2990,00	1186,00	446,00					635,00					0,70
AL033	2960,00	3406,00	474,00	29,00	119,00	328,00	16,00	2779,00	74,00	86,00	1517,00	1102,00	0,17
AL034	620,00	234,00	70,00	3,00	16,00	51,00	0,00	165,00	4,00	5,00	101,00	55,00	0,42
AL035	1310,00	694,00	282,00	65,00	53,00	163,00	0,00	282,00	20,00	0,00	182,00	78,00	1
AL036	910,00	595,00	369,00	15,00	88,00	265,00	5,00	221,00	2,00	3,00	62,00	154,00	1,67
AL037	900,00	420,00	65,00	5,00	13,00	47,00	1,00	369,00	41,00	10,00	72,00	246,00	0,17
AL038	690,00	425,00	63,00	6,00	13,00	44,00	5,00	356,00	4,00	5,00	99,00	249,00	0,18
AL039	410,00	175,00	60,00	13,00	17,00	30,00	0,00	104,00	9,00	2,00	75,00	18,00	0,58
AL040	700,00	464,00	199,00	22,00	81,00	97,00	D	226,00	62,00	10,00	79,00	76,00	0,88
AL041	870,00	545,00	140,00	19,00	15,00	106,00	6,00	396,00	13,00	D	108,00	272,00	0,36
