# Supplementary material for: Torque Teno Virus Viral Load as a Marker of Immune Function in Allogeneic Haematopoietic Stem Cell Transplantation Recipients
Source: Viruses. 2020 Nov 11;12(11):1292. doi: 10.3390/v12111292 (PMC7696489; doi:10.3390/v12111292)
Supplement: Supplementary file 1 [file viruses-12-01292-s001.zip › viruses-980283-supplementary/Table S2.pdf]

viruses

MDPI

Table S2. Comparison of TTV plasma viral load with clinical characteristics of 41 allogeneic haematopoietic stem cell transplant recipients. Comparison of median [IQR] TTV plasma viral load with clinical datas was performed using Mann-Whitney test (***. p< 0.001). #Viral infection/reactivation include Astrovirus, Adenovirus, BK virus, Cytomegalovirus, Epstein-Barr virus, Human Herpesvirus 6, Herpes Simplex 1 virus, Metapneumovirus, Picornavirus, Hepatitis E virus and Varicella-Zoster virus.

	**n (%)**	**TTV Log copy/mL median [IQR]**	***p-value***
Sex			
Male	24 (59)	3.7 [2.9-4.8]	0.38
Female	17 (41)	4.1 [3.5-4.4]	
Underlying haematological diseases			
Myeloid neoplasm and acute leukaemia	37 (90)	3.9 [3.1-4.4]	0.28
Mature lymphoid. histiocytic. and dendritic neoplasms	4 (10)	5.0 [3.8-5.8]	
Donor types			
Matched related	23 (56)	3.9 [3.1-4.6]	0.52
Unrelated	18 (44)	4.1 [3.2-4.7]	
Stem cell sources			
Peripheral blood cells	28 (68)	4.0 [3.1-4.8]	0.43
Bone marrow	13 (32)	3.7 [3.2-4.1]	
Conditioning regimen			
MAC	17 (41)	3.6 [2.9-4.2]	0.22
RIC	24 (59)	4.1 [3.1-4.9]	
TBI			
Yes	11 (27)	3.7 [2.9-4.5]	0.46
No	30 (73)	4.1 [3.1-4.6]	
GVHD			
Yes	31 (76)	3.7 [3.0-4.8]	0.40
No	10 (24)	4.1 [3.8-4.3]	
Immunosuppressive therapy			
Yes	32 (78)	3.9 [3.0-4.4]	0.54
No	9 (22)	4.0 [3.2-4.9]	
IVIG infusion			
Yes	23 (56)	3.9 [3.0-4.5]	0.59
No	18 (44)	4.1 [3.2-4.7]	
#Viral opportunistic infection/reactivation during post-transplant period			
Yes	26 (63)	4.1 [3.5-4.9]	0.02
No	15 (37)	3.2 [2.8-4.0]	
CMV virus infection/reactivation during post-transplant period			
Yes	9 (22)	4.8 [4.0-5.9]	0.02
No	32 (78)	3.7 [2.9-4.3]	

Abbreviations: CMV. cytomegalovirus; GvHD. graft-versus-host disease; IQR. interquartile; IVIG. intravenous immunoglobulins; MAC. myeloablative conditioning; RIC. reduced intensity conditioning; TBI. total body irradiation; TTB. torque teno virus.
